# Multivariate analyses of molecular genetic associations between childhood psychopathology and adult mood disorders and related traits

**DOI:** 10.1002/ajmg.b.32922

**Published:** 2022-11-15

**Authors:** Wonuola A. Akingbuwa, Anke R. Hammerschlag, Andrea G. Allegrini, Hannah Sallis, Ralf Kuja-Halkola, Kaili Rimfeld, Paul Lichtenstein, Sebastian Lundstrom, Marcus R. Munafò, Robert Plomin, Michel G. Nivard, Meike Bartels, Christel M. Middeldorp

**Affiliations:** 1 Department of Biological Psychology, Vrije Universiteit Amsterdam, Amsterdam, The Netherlands; 2 Amsterdam Public Health Research Institute, Amsterdam, The Netherlands; 3 Child Health Research Centre, the University of Queensland, Brisbane, Queensland, Australia; 4 Social, Genetic and Developmental Psychiatry Centre, Institute of Psychiatry, Psychology and Neuroscience, King's College London, London, UK; 5 Division of Psychology and Language Sciences, Department of Clinical, Educational and Health Psychology, University College London, London, UK; 6 School of Psychological Science, University of Bristol, Bristol, UK; 7 MRC Integrative Epidemiology Unit, University of Bristol, Bristol, UK; 8 Centre for Academic Mental Health, Population Health Sciences, Bristol Medical School, University of Bristol, Bristol, UK; 9 Department of Medical Epidemiology and Biostatistics, Karolinska Institutet, Stockholm, Sweden; 10 Centre for Ethics Law and Mental Health, Gillberg Neuropsychiatry Centre, University of Gothenburg, Gothenburg, Sweden; 11 NIHR Biomedical Research Centre, University Hospitals Bristol NHS Foundation Trust, University of Bristol, Bristol, UK; 12 Child and Youth Mental Health Service, Children's Health Queensland Hospital and Health Services, Brisbane, Queensland, Australia

**Keywords:** childhood psychopathology, major depression, multivariate regression, polygenic scores

## Abstract

Ubiquitous associations have been detected between different types of childhood psychopathology and polygenic risk scores based on adult psychiatric disorders and related adult outcomes, indicating that genetic factors partly explain the association between childhood psychopathology and adult outcomes. However, these analyses in general do not take into account the correlations between the adult trait and disorder polygenic risk scores. This study aimed to further clarify the influence of genetic factors on associations between childhood psychopathology and adult outcomes by accounting for these correlations. Using a multivariate multivariable regression, we analyzed associations of childhood attention-deficit/hyperactivity disorder (ADHD), internalizing, and social problems, with polygenic scores (PGS) of adult disorders and traits including major depression, bipolar disorder, subjective well-being, neuroticism, insomnia, educational attainment, and body mass index (BMI), derived for 20,539 children aged 8.5–10.5 years. After correcting for correlations between the adult phenotypes, major depression PGS were associated with all three childhood traits, that is, ADHD, internalizing, and social problems. In addition, BMI PGS were associated with ADHD symptoms and social problems, while neuroticism PGS were only associated with internalizing problems and educational attainment PGS were only associated with ADHD symptoms. PGS of bipolar disorder, subjective well-being, and insomnia were not associated with any childhood traits. Our findings suggest that associations between childhood psychopathology and adult traits like insomnia and subjective well-being may be primarily driven by genetic factors that influence adult major depression. Additionally, specific childhood phenotypes are genetically associated with educational attainment, BMI and neuroticism.

## 1 Introduction

Psychiatric disorders cause significant distress and impaired functioning. They are also highly comorbid, with extensive phenotypic and symptom overlap. Comorbidity and symptom overlap has been observed between a range of disorder types including mood disorders like depression and anxiety (Johansson, Carlbring, Heedman, Paxling, & Andersson, 2013; Tiller, 2013), childhood-onset neurodevelopmental disorders like attention-deficit/hyperactivity disorder (ADHD), autism spectrum disorder (ASD), and Tourette syndrome (Huisman-van Dijk, Schoot, Rijkeboer, Mathews, & Cath, 2016), as well as between ADHD and anxiety disorders and depression (D’Agati, Curatolo, & Mazzone, 2019; Gnanavel, Sharma, Kaushal, & Hussain, 2019). Importantly, a substantial proportion of children and adolescents with psychopathology continue to have psychiatric disorders in adulthood, as well as poorer outcomes related to physical health and functional outcomes, including higher body mass index (BMI), and lower educational attainment among others (Asselmann, Wittchen, Lieb, & Beesdo-Baum, 2018; Copeland, Alaie, Jonsson, & Shanahan, 2021; Costello & Maughan, 2015; McLeod, Horwood, & Fergusson, 2016; Oerlemans, Wardenaar, Raven, Hartman, & Ormel, 2020; Ormel et al., 2017). Thus, psychopathology traits are correlated with each other, and are linked to increased risk for negative outcomes, both related to mental health and beyond.

Using both twin- and molecular-based analyses, studies have shown genetic influences on the stability and continuity of psychopathology traits including attention problems, anxiety, and depression over time. Indeed, there is evidence of genetic influence both for homotypic continuity (when a disorder is predicted by itself at a later time point) and heterotypic continuity (when one disorder predicts another at a later time point, e.g., childhood anxiety is associated with schizophrenia later in life) (Akingbuwa et al., 2020; Hannigan, Walaker, Waszczuk, McAdams, & Eley, 2017; Kan et al., 2013; Nivard et al., 2015; Sallis et al., 2017; Stergiakouli et al., 2017). Many studies investigating such genetic associations between childhood psychopathology and adult phenotypes have employed polygenic scores (PGS), which index an individual’s genetic risk for a trait based on previously determined effect sizes for alleles associated with the trait (Wray et al., 2014). They have been used to show that shared genetic overlap likely underlies associations between childhood psychopathology and adult mood disorders including depression and anxiety, as well as related traits like neuroticism, insomnia, and subjective well-being (Akingbuwa et al., 2020; Kwong et al., 2021). Furthermore, PGS have also been used to demonstrate genetic overlap between childhood psychopathology and mood disorder-related functional outcomes, such as educational attainment, and BMI (Akingbuwa et al., 2020; Jansen et al., 2018; Stergiakouli, Smith, et al., 2017).

Crucially, these associations are typically analyzed in univariate analyses. However, the adult traits are phenotypically and genetically correlated (Anttila et al., 2018; Baselmans et al., 2019; Caspi et al., 2014; P. H. Lee et al., 2019). This raises the question of whether the ubiquitous genetic associations observed are genuine or whether they are driven by unaccounted correlations between related traits. Knowledge of how underlying correlations influence genetic associations may provide insight into trans-diagnostic continuity of psychopathology across the lifespan and can be of importance for building prediction models for outcomes of childhood psychopathology.

In the current study, we performed a preregistered (https://osf.io/7nkw8) multivariate analysis to investigate genetic associations between childhood psychopathology symptoms and adult depression and related traits. In previous analyses, we observed associations between PGS of major depression and childhood ADHD symptoms, internalizing, and social problems using univariate analyses. Depression-related traits including BMI, neuroticism, and insomnia, among others, were also shown to be genetically associated with childhood psychopathology (Akingbuwa et al., 2020). In the current analyses, we were interested in exploring how accounting for the correlations between the adult trait and disorder PGS affects these previously observed univariate genetic associations between them. We obtained maternal-rated data for 20,539 children across three cohorts. As previous analyses largely showed no age effects in associations between childhood psychopathology and PGS of adult phenotypes, we focused the current analysis at the age at which we had the most combined data, which was at age 9–10.

## 2 Methods

### 2.1 Participants and measures

Maternal-rated measures of ADHD symptoms, internalizing, and social problems were obtained for children aged 9–10 years from four population-based cohorts including the Avon Longitudinal Study of Parents and Children (ALSPAC; Boyd et al., 2013; Fraser et al., 2013; Northstone et al., 2019), Child and Adolescent Twin Study in Sweden (CATSS; Anckarsäter et al., 2012), Netherlands Twin Register (NTR; Ligthart et al., 2019), and Twins Early Development Study (TEDS; Rimfeld et al., 2019; Table 1). CATSS, NTR, and TEDS are population based twin cohorts while ALSPAC is a population based birth cohort that recruited all pregnant women in the former county of Avon with an expected due date between April 1991 and December 1992 Childhood psychopathology was measured in ALSPAC and TEDS using the hyperactivity-inattention, emotional symptoms, and peer relationship problems subscales of the Strength and Difficulties Questionnaire (SDQ; Goodman, 1997), while in the NTR, the attention, internalizing, and social problems subscales of the Child Behavior Checklist (CBCL; Achenbach, 2014) were used. In CATSS, the AD/HD module of the Autism-Tics, AD/HD, and other comorbidities inventory (Larson et al., 2010), was used to measure ADHD symptoms. For internalizing problems, the Screen for Child Anxiety Related Emotional Disorders (SCARED; Birmaher et al., 1997) was selected over the Short Mood and Feelings Questionnaire (SMFQ; Sharp, Goodyer, & Croudace, 2006). This is because while they both had comparable psychometric properties, the SCARED measures symptoms over the past 3 months, which is more in line with the longer-term measures of the CBCL (2 months) and SDQ (6 months) used by other cohorts, compared to the SMFQ which measures symptoms over the past 2 weeks. The CATSS cohort did not have a measure of social problems at age 9–10.

Genotyping and quality control were performed by each cohort according to common standards and have been previously described (Akingbuwa et al., 2020). We obtained PGS for disorder and traits including major depression (N. R. Wray et al., 2018), bipolar disorder (Stahl et al., 2019), subjective well-being, neuroticism (Okbay et al., 2016), insomnia (Hammerschlag et al., 2017), educational attainment (J. J. Lee et al., 2018), and BMI (Yengo et al., 2018), calculated using LDpred (Vilhjálmsson et al., 2015). LDpred allows the inclusion of prior probabilities which correspond to the assumed proportion of genetic variants thought to be causal for a given phenotype. We used PGS at the most predictive priors per phenotype, determined from previous univariate analyses (Akingbuwa et al., 2020). All GWAS discovery samples consisted of adult only samples, with the exception of major depression which had a small proportion of adolescent samples. GWAS discovery sample sizes for each phenotype are included in Table S1. Data collection was approved by each cohort’s local institutional review or ethics board, waiving the need for informed consent for this study. Analyses were limited to individuals of European ancestry.

### 2.2 Statistical analyses

**Figure 1:**
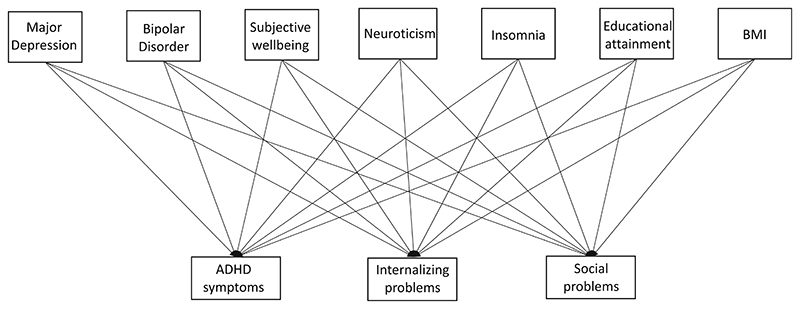
This figure includes only main predictor and outcome measures but does not include various covariate accounted for in the regression model. BMI, body mass index; ADHD, attention deficit hyperactivity disorder

The main model tested is described in Figure 1. The model represents a multivariate regression with three dependent and seven independent variables, as well as additional covariates. The dependent variables are the maternal-rated measures of ADHD symptoms, internalizing, and social problems, while the independent variables are PGS of major depression, bipolar disorder, subjective well-being, neuroticism, insomnia, educational attainment, and BMI. Multivariate multivariable regression analyses were performed in R using path specification in the OpenMx package (Boker et al., 2020; Hunter, 2018; Neale et al., 2016; Pritikin, Hunter, & Boker, 2015). Full information maximum likelihood (FIML) estimation (Enders & Bandalos, 2001), optimized in OpenMx was used to account for missingness in the outcome (childhood measures) data. We also accounted for the effects of sex, age, genetic principal components (to correct for population stratification), genotyping chip, and batch effects on the childhood measures, by including them as covariates in the model (Table 1). Given that our previous analyses in Akingbuwa et al. (2020) showed no differences in effects across types of childhood psychopathology measures, we did not include measurement scale as a covariate in the current analyses.

Both the childhood measures and the PGS were scaled so that they each had a mean of zero and *SD* of 1. This allowed for data to be jointly analyzed across cohorts using a multi-group model, which aggregates fit statistics from separate submodels specified for each cohort. Correlations and regression coefficients were constrained to be equal across cohorts, while estimates for the PCs, genotyping chip and batch effects, as well as their variances which were estimated separately per cohort. We corrected for relatedness in the twin samples (CATSS, NTR, TEDS) by estimating the cross-twin covariance for each outcome measure, as well as cross-twin cross-trait covariances.

We adjusted our significance threshold to account for multiple testing, using Bonferroni adjustment (*α* = 0.05/number of tests), where the number of tests is the number of outcome measures multiplied by the number of predictors (*α* = 0.05/[3 × 7] = 0.00238).

## 3 Results

Across all cohorts, 20,539 children were included in the current analyses. Their ages ranged from 8.5 to 10.5 years. Full descriptive statistics per cohort for age and childhood measures, as well as sex-based information are provided in Tables S2 and S3.

### 3.1 Associations between adult trait and disorder PGS and childhood traits

We fitted a multivariate multivariable regression model investigating associations between the three childhood outcome measures, and PGS at a prior of 0.75 for educational attainment and BMI, 0.5 for major depression, and insomnia, 0.3 for neuroticism, 0.1 for bipolar disorder, and 0.03 for subjective well-being. Negative correlations between the PGS ranged from –0.009 to –0.305 while positive correlations ranged from 0.011 to 0.306 (Table 2). The pattern of correlations between the adult trait and disorder PGS was similar to those seen in previous analyses, with high correlations between variables on the depression-well-being spectrum including neuroticism, and lower associations with other phenotypes like BMI, educational attainment and bipolar disorder (Anttila et al., 2018; Hammerschlag et al., 2017; Jansen et al., 2019; Okbay et al., 2016). Further, insomnia, subjective well-being, and neuroticism were also correlated with each other, although to a slightly lesser extent.

**Figure 2:**
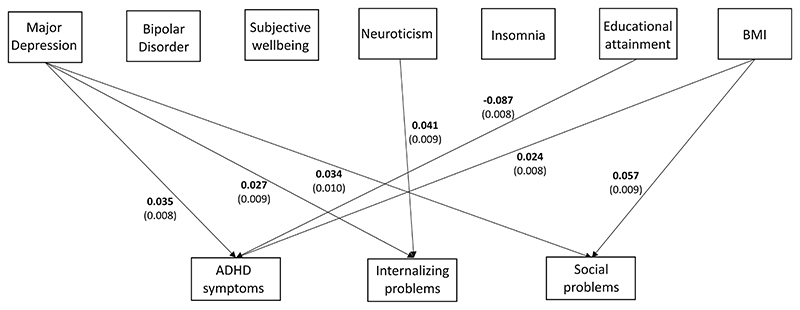
Path coefficients from multivariate model showing significant associations between PGS of adult phenotypes and childhood psychopathology measures. Standard errors of association estimates are in brackets. ADHD, attention deficit hyperactivity disorder; BMI, body mass index

After correction for multiple testing (*α* = .00238), we observed significant positive associations between BMI PGS and ADHD symptoms (*β* = .024, 95% CI = 0.008–0.039, SE = .008, *p* = .002) and social problems (*β* = .057, 95% CI = 0.039–0.076, SE = .009, *p* = 1.37 × 10^–09^), between major depression PGS and ADHD symptoms (*β* = .035, 95% CI =0.019–0.051, SE = .008, *p* = 2.23 × 10^–05^), internalizing (*β* = .027, 95% CI = 0.010–0.044, SE = .009, *p* = .002), and social problems (*β* = .034, 95% CI =0.014–0.053, SE = .010, *p* = .001), and finally between neuroticism and internalizing problems (*β* = .041, 95% CI =0.024–0.059, SE = .009, *p* = 4.97 × 10^–06^). We also observed significant negative associations between educational attainment PGS and ADHD symptoms (*β* = –.087[95% CI = –0.071 to –0.102], SE = .008, *p* = 2.45 × 10^–28^) (Figure 2). Other associations between childhood measures and PGS were not statistically significant (Table 3).

## 4 Discussion

So far, studies have primarily used univariate analyses to investigate genetic associations between childhood psychopathology and PGS of adult mood disorders and related traits like neuroticism, insomnia and subjective-well-being, as well as functional outcomes like educational attainment and BMI (Akingbuwa et al., 2020). In the current study, we follow-up previous univariate findings with a multivariate multivariable regression analysis with the aim of exploring how underlying correlations between these variables influences the strength/presence of previously observed associations. Using a multivariate model, we accounted for correlations between the PGS of adult traits and disorders. We found that major depression PGS were significantly associated with all three measures of childhood psychopathology. In addition, BMI PGS were positively associated with ADHD symptoms and social problems, and neuroticism PGS were positively associated with internalizing problems, while educational attainment PGS were negatively associated with ADHD symptoms. These results suggest associations between these adult trait and disorder PGS and childhood psychopathology, over and above the effect of any correlations with other adult phenotype PGS. Previously reported associations of childhood psychopathology with PGS of insomnia, neuroticism, and subjective well-being were largely no longer present.

We observed differential genetic associations between childhood psychopathology and adult traits and disorders, with all childhood problems investigated associated with genetic risk for major depression. On the other hand, genetic risk for traits like neuroticism, educational attainment and BMI appeared to be related to specific childhood psychopathology measures. The nonspecific association of childhood psychopathology with depression PGS suggests that there are genetic variants associated with depression and shared across the three childhood traits, which might be indicative of a dimensional structure of psychopathology where any type of childhood psychopathology is linked to genetic risk for depression. Although it is also possible that another unmeasured factor or trait is associated with all three childhood psychopathology measures and depression, which explains the shared genetic risk.

To some extent, we observed a similar pattern for PGS of BMI as for PGS of depression, in that it showed associations with social problems and ADHD symptoms, that is, there are genetic variants associated with BMI which are shared with both traits. However we did not observe this with PGS of educational attainment, and neuroticism, which were associated with only ADHD symptoms and internalizing problems, respectively. This indicates that there are also specific genetic factors that are associated with educational attainment and ADHD symptoms, and with neuroticism and internalizing problems, which are not shared with the other childhood traits. This is despite the fact that we observed modest correlations between the childhood traits (Figure 2).

These findings highlight the importance of both general and unique genetic factors to the understanding of psychiatric etiology. Moreover, these results also suggest that many of the previously detected genetic associations between childhood traits and PGS of adult depression-related traits may be the result of their genetic correlations with depression (Akingbuwa et al., 2020). An exception was neuroticism PGS, which were still associated with internalizing symptoms. Additionally, we observed no associations between bipolar disorder and childhood psychopathology, despite the fact that bipolar disorder also shows moderate genetic correlations with major depression (Anttila et al., 2018). This may be due to a lack of power in the bipolar disorder discovery GWAS.

We showed that the use of multivariate methodology is important in furthering our understanding of genetic mechanisms underlying psychopathology across childhood and adulthood, but also associations between childhood psychopathology traits and functional outcomes in adulthood. Importantly, genetic risk for depression appeared to be linked to a myriad of childhood psychopathology traits, suggesting shared heritability across development. While this is perhaps expected for associations with internalizing problems, observed cross-disorder associations between major depression PGS and ADHD and social problems have implications for trans-diagnostic continuity across development. It contests the view of psychiatric traits or disorders as enduring discreet conditions, and raises clinically important questions as to the validity of distinct diagnostic boundaries. The observed substantial phenotypic correlations between the childhood traits may hint at symptom overlap, while nonspecific associations with depression suggest shared genetic risk for them. Neither of these is strongly supportive of categorical classifications of psychopathology.

The observed associations may also be indicative of a causal association between childhood measures and depression in adulthood, which warrants future analyses of causality. The independent effect of neuroticism PGS on internalizing problems, on top of the effect of PGS for major depression is also interesting in this regard. It could be speculated that the measurement of internalizing problems in childhood is more reflective of a trait of emotional instability just like neuroticism, than of a depressive state like major depression. Furthermore, in conceptualizing causal factors underlying comorbidity between childhood psychopathology, negative emotionality (also known as neuroticism) has been proposed to be a common feature underlying all childhood psychopathology (Lahey, Krueger, Rathouz, Waldman, & Zald, 2017; Rhee, Lahey, & Waldman, 2015). Interestingly, we only observe associations between neuroticism PGS and internalizing problems. However, the nature of PGS is such that the variance that they explain is very small. This means that it is likely/certain that associations observed do not reflect the total genetic overlap between neuroticism and childhood psychopathology. Replication of this result with PGS from larger GWAS is necessary.

Our findings regarding educational attainment and BMI replicate well established findings for genetic overlap between reduced educational attainment and ADHD symptoms in childhood (de Zeeuw et al., 2014; Jansen et al., 2018; Stergiakouli, Martin, et al., 2017), as well as for BMI and childhood psychopathology, particularly ADHD (Anttila et al., 2018; Du Rietz et al., 2018). Genetic analyses of causal mechanisms between ADHD and BMI have so far been inconclusive, with evidence of causality in both directions (Leppert et al., 2021; Liu et al., 2020; Martins-Silva et al., 2019). Analyses of causality between ADHD and educational attainment are fewer still, with one study showing evidence of bidirectional causal associations (Dardani et al., 2021). We add to the growing body of literature supporting associations between genetic risk for psychopathology, and health and sociodemographic outcomes in later life. The effect sizes reported were generally quite small which perhaps suggest that interpretations of our findings should be made cautiously. Nevertheless, more studies with a focus on causality are crucial, as knowledge of causal mechanisms may eventually inform clinical interventions, as well as risk for adverse effects of functional outcomes in the long-term.

Our study had some limitations. PGS analyses have been shown to include the effects of passive gene–environment correlation—an association between a child’s genotype and familial environment as result of parents providing environments that are influenced by their own genotypes (Selzam et al., 2019), which are unaccounted for in the present study and may have affected our findings. Second, while PGS involve aggregating the effects of many trait-associated variants, they are not informative about which specific genetic variants drive the observed associations and further fine-mapping and variant prioritization analyses are required to shed more light on this. Further, the small proportion of variance explained by the PGS means that they are currently unable to be used clinically. However, the aim of the current study was primarily to investigate the underlying genetic architecture. Finally, the case samples from the major depression GWAS used to construct the PGS in the current study were ascertained using minimal phenotyping. Minimal phenotyping involves leveraging information from sources including hospital registers, self-reported symptoms, help seeking, or medication, in order to maximize statistical power to detect genetic variants. Major depression defined through minimal phenotyping has been shown to have different genetic architecture from strictly/clinically defined major depressive disorder (MDD), with genetic loci that are not specific to MDD (Cai et al., 2020). Therefore, our findings regarding major depression may be a function of the nonspecific nature of genetic factors associated with minimally phenotyped depression. However, major depression defined in this manner shows strong correlation with MDD, as well as good PGS-based prediction of MDD in independent samples (Cai et al., 2020; N. R. Wray et al., 2018). Nevertheless, similar analyses using clinical measures of MDD are important to further confirm our findings.

Results from this study show differential genetic associations between childhood psychopathology and adult depression and related traits, which may be suggestive of both shared and unique genetic factors underlying these associations. Future studies combining multivariate methodology with molecular data should focus on further unraveling these effects not just for psychopathology traits, but also associated functional and nonpsychiatric outcomes such as educational attainment and BMI.

## Supplementary Material

Supplementary information

## Figures and Tables

**Table 1:** Sample characteristics

Cohort	Phenotype(s) measured	Scale(s)	Sample size	Covariates included in regression model
ALSPAC	ADHD symptoms, internalizing problems, social problems	SDQ	5,025	10 genetic PCs, age, sex
CATSS	ADHD symptoms, internalizing problems	A-TAC, SCARED	7,284	10 genetic PCs, sex
NTR	ADHD symptoms, internalizing problems, social problems	ASEBA-CBCL	3,652	10 genetic PCs, genotyping chip, age, sex
TEDS	ADHD symptoms, internalizing problems, social problems	SDQ	4,578	10 genetic PCs, genotyping chip, genotyping batch, age, sex

Abbreviations: ALSPAC, Avon Longitudinal Study of Parents and Children; ASEBA, Achenbach System of Empirically Based Assessment (Achenbach, 2014); A-TAC, Autism-Tics, AD/HD and other comorbidities inventory (Larson et al., 2010); CATSS, Child and Adolescent Twin Study in Sweden; CBCL, Child Behavior Checklist (Achenbach, 2014); NTR, Netherlands Twin Register; PCs, principal components; SCARED, Screen for Child Anxiety Related Emotional Disorders (Birmaher et al., 1997); SDQ, Strength and Difficulties Questionnaire (Goodman, 1997); TEDS, Twins Early Development Study.

**Table 2:** Polygenic scores correlation matrix

	Major depression	Bipolar disorder	Subjective wellbeing	Neuroticism	Insomnia	Educational attainment	BMI
Major depression	1	0.184	–0.215	0.306	0.191	–0.125	0.05
Bipolar disorder	0.184	1	–0.03	0.068	0.014	0.068	–0.009
Subjective well-being	–0.215	–0.03	1	–0.305	–0.118	0.047	0.011
Neuroticism	0.306	0.068	–0.305	1	0.244	–0.152	–0.082
Insomnia	0.191	0.014	–0.118	0.244	1	–0.152	0.04
Educational attainment	–0.125	0.068	0.047	–0.152	–0.152	1	–0.201
BMI	0.05	–0.009	0.011	–0.082	0.04	–0.201	1

Note: Matrix represents the average correlation between the scaled PGS of the adult phenotypes across four cohorts.

**Table 3:** Results from multivariate regression model

	ADHD symptoms				Internalizing problems				Social problems			
PGS (discovery sample size)	*β* (SE)	*p* value	ci.lb	ci.ub	*β* (SE)	*p* value	ci.lb	ci.ub	*β* (SE)	*p* value	ci.lb	ci.ub
Major depression (173,005)	**0.035 (0.008)**	**2.23 × 10^–05^**	**0.019**	**0.051**	**0.027 (0.009)**	**.002**	**0.010**	**0.044**	**0.034 (0.010)**	**.001**	**0.014**	**0.053**
Bipolar disorder (51,710)	–0.002 (0.008)	.743	–0.018	0.013	–0.004(0.008)	.626	–0.020	0.012	0.009 (0.009)	.330	–0.009	0.028
Subjective well-being (298,420)	0.004 (0.008)	.639	–0.012	0.019	–0.002 (0.009)	.832	–0.019	0.015	–0.006 (0.010)	.561	–0.025	0.013
Neuroticism (170,911)	0.004 (0.008)	.614	–0.012	0.021	**0.041 (0.009)**	**4.97 × 10^–06^**	**0.024**	**0.059**	0.029 (0.010)	.004	0.009	0.049
Insomnia (113,006)	0.008 (0.008)	.334	–0.008	0.023	–0.004 (0.008)	.610	–0.021	0.012	0.002 (0.010)	.826	–0.017	0.021
Educational attainment (766,345)	–0.087 (0.008)	**2.45 × 10^–28^**	**0.102**	**0.071**	–0.025 (0.009)	.003	–0.042	–0.009	–0.015 (0.010)	.129	–0.034	0.004
BMI (681,275)	**0.024 (0.008)**	**.002**	**0.008**	**0.039**	0.011 (0.008)	.190	–0.005	0.027	**0.057 (0.009)**	**1.37 × 10^–09^**	**0.039**	**0.076**
Sex	**0.210 (0.009)**	**7.43 × 10^–115^**	**0.192**	**0.228**	**–0.081 (0.010)**	**1.99 × 10^–16^**	**–0.100**	**–0.061**	**0.074 (0.011)**	**3.40 × 10^–11^**	**0.052**	**0.096**
Age	–0.010 (0.009)	.294	–0.028	0.009	0.005 (0.010)	.584	–0.014	0.024	–0.003 (0.010)	.776	–0.022	0.017

*Note:* Estimates for all model constrained variables, *β* (SE), estimate of regression association and accompanying standard error from multivariate model; ci.lb, lower bound of 95%Cl; ci.ub, upper bound of 95% Cl. Bold estimates represent significant associations at Bonferroni-corrected threshold. Assessment of overall model fit suggested an acceptable to good fit based on RMSEA (0.047) but not CFI (–0.075) and TLI (–0.013).

## Data Availability

The data that support the findings from this study are available from the different cohorts involved. Restrictions apply to the availability of these data as they include individual level genetic and phenotypic data. Data are available on successful application to the relevant cohort.
