# Supplementary material for: Multivariate analyses of molecular genetic associations between childhood psychopathology and adult mood disorders and related traits
Source: Am J Med Genet B Neuropsychiatr Genet. Author manuscript; Available in PMC 2023 Sep 1. (PMC7615008; doi:10.1002/ajmg.b.32922)
Supplement: Supplementary information [file EMS185054-supplement-Supplementary_information.docx]

Multivariate analyses of molecular genetic associations between childhood psychopathology and adult mood disorders and related traits.

Akingbuwa et al

# **Cohort Funding and Acknowledgements**

We are grateful to all families and participants who took part in these studies. We also acknowledge and appreciate the unique efforts of the research teams and practitioners contributing to the collection of this wealth of data.

**ALSPAC**

The Avon Longitudinal Study of Parents and Children (ALSPAC) is a longitudinal birth cohort which aimed to recruit all pregnant women in the former county of Avon in the United Kingdom (UK) with a due date between April 1991 and December 1992. Detailed information has continued to be collected on mothers, partners and children in this cohort, details of which have been previously described elsewhere ^1-3^. Ethical approval for the study was obtained from the ALSPAC Ethics and Law Committee and the Local Research Ethics Committees. Consent for biological samples has been collected in accordance with the Human Tissue Act (2004). Informed consent for the use of data collected via questionnaires and clinics was obtained from participants following the recommendations of the ALSPAC Ethics and Law Committee at the time. Please note that the study website contains details of all the data that is available through a fully searchable data dictionary and variable search tool <http://www.bris.ac.uk/alspac/researchers/data-access/data-dictionary/>.

We are extremely grateful to all the families who took part in this study, the midwives for their help in recruiting them, and the whole ALSPAC team, which includes interviewers, computer and laboratory technicians, clerical workers, research scientists, volunteers, managers, receptionists and nurses. The UK Medical Research Council and Wellcome (Grant ref: 217065/Z/19/Z) and the University of Bristol provide core support for ALSPAC. This publication is the work of the authors and

Wonuola Akingbuwa and Christel Middeldorp will serve as guarantors for the contents of this paper. A comprehensive list of grants funding is available on the ALSPAC website (http://www.bristol.ac.uk/alspac/external/documents/grant-acknowledgements.pdf). GWAS data was generated by Sample Logistics and Genotyping Facilities at Wellcome Sanger Institute and LabCorp (Laboratory Corporation of America) using support from 23andMe. This study was supported by the National Institute for Health Research (NIHR) Biomedical Research Centre at the University Hospitals Bristol National Health Service Foundation Trust and the University of Bristol. The views expressed in this publication are those of the author(s) and not necessarily those of the National Health Service, the National Institute for Health Research or the Department of Health.

**CATSS**

The Child and Adolescent Twin Study in Sweden (CATSS) is a longitudinal twin study targeting all twins born in Sweden since July 1, 1992. Parents are interviewed regarding their children’s mental health and behaviours in connection with their 9^th^ and 12^th^ birthdays, and at age 15 and 18, both twins and parents provide this information ^4^. The CATSS has ethical approval from the Regional Ethical Review Board of Stockholm and all participants and parents provide consent.

The Child and Adolescent Twin Study in Sweden is supported by Swedish Research Council (Medicine, Humanities and Social Science, and SIMSAM), Funds under the ALF agreement, and the Swedish Research Council for Health, Working Life and Welfare (FORTE). The research leading to these results has also received funding from the European Union Seventh Framework Programme (FP7/2007-2013) under grant agreement no 602768. CATSS is a part of the Swedish Twin Registry, managed by Karolinska Institutet and receiving funding through the Swedish Research Council under the grant no 2017-00641.

**NTR**

The Netherlands Twin Register (NTR) has collected data on twins and their families, as well as families with new-born twins and triplets since 1987 ^5^. The young NTR (YNTR) is made up of twins registered at birth by their parents. Information on twins under 14 years are provided by their parents and teachers, and from age 14 onwards, data collection is via self-report. Ethical approval was provided by the Central Ethics Committee on Research Involving Human Subjects of the VU University Medical Center, Amsterdam, an Institutional Review Board certified by the U.S. Office of Human Research Protections (IRB number IRB-2991 under Federal-wide Assurance-3703; IRB/institute codes 94/105, 96/205, 99/068, 2003/182, 2010/359) and participants provided informed consent.

Data collection in the NTR was supported by NWO: Twin-family database for behavior genetics and genomics studies (480-04-004); “Spinozapremie” (NWO/SPI 56-464-14192; “Genetic and Family influences on Adolescent psychopathology and Wellness” (NWO 463-06-001); “A twin-sib study of adolescent wellness” (NWO-VENI 451-04-034); ZonMW “Genetic influences on stability and change in psychopathology from childhood to young adulthood” (912-10-020); “Netherlands Twin Registry Repository” (480-15-001/674); “Biobanking and Biomolecular Resources Research Infrastructure” (BBMRI –NL (184.021.007 and 184.033.111). We acknowledge FP7-HEALTH-F4-2007, grant agreement no 201413 (ENGAGE), and the FP7/2007-2013 funded ACTION (grant agreement no 602768) and the European Research Council (ERC-230374). Part of the genotyping was funded by the Genetic Association Information Network (GAIN) of the Foundation for the National Institutes of Health, Rutgers University Cell and DNA Repository (NIMH U24 MH068457-06), the Avera Institute, Sioux Falls, South Dakota (USA) and the National Institutes of Health (NIH R01 HD042157-01A1, MH081802, Grand Opportunity grants 1RC2 MH089951 and 1RC2 MH089995).

**TEDS**

The Twins Early Development Study (TEDS) is a longitudinal sample of twins born in England and Wales between 1994 and 1996^6^. Families were invited to take part in studies at various stages when the twins were aged 2 to 16 years. Informed consent is always obtained prior to collecting data. Ethical approval for this study was received from King’s College London Ethics Committee (Ref PNM/09/10-104).

We gratefully acknowledge the ongoing contribution of the participants in the Twins Early Development Study (TEDS) and their families. TEDS is supported by a program grant to RP from the UK Medical Research Council (MR/M021475/1 and previously G0901245), with additional support from the US National Institutes of Health (AG046938).

**Supplementary Table 1. GWAS sample sizes**

| **Trait (reference)** | **Sample size (case/control)** |
| --- | --- |
| Major depression^7^ | 173,005 (59,851/113,154) |
| Bipolar disorder^8^ | 51,710 (20,352/31,358) |
| Subjective well-being^9^ | 298,420 |
| Neuroticism^9^ | 170,911 |
| Insomnia^10^ | 113,006 (32,384/80,622) |
| Educational attainment^11^ | 766,345 |
| BMI^12^ | 681,275 |
| Height^12^ | 693,529 |

**Supplementary Table 2. Sample age descriptive statistics**

|  | All | | | | | Males | | | | | Females | | | | |
| --- | --- | --- | --- | --- | --- | --- | --- | --- | --- | --- | --- | --- | --- | --- | --- |
| Cohort | **N** | **Mean** | **SD** | **Min** | **Max** | **N** | **Mean** | **SD** | **Min** | **Max** | **N** | **Mean** | **SD** | **Min** | **Max** |
| ALSPAC | 5025 | 9.64 | 0.11 | 9.50 | 10.42 | 2544 | 9.64 | 0.11 | 9.50 | 10.42 | 2481 | 9.64 | 0.11 | 9.50 | 10.42 |
| CATSS* | 7284 | 9.00 | 0.00 | 9.00 | 9.00 | 3636 | 9.00 | 0.00 | 9.00 | 9.00 | 3648 | 9.00 | 0.00 | 9.00 | 9.00 |
| NTR | 3652 | 9.89 | 0.37 | 8.50 | 10.50 | 1726 | 9.90 | 0.38 | 8.50 | 10.50 | 1926 | 9.89 | 0.36 | 8.50 | 10.5 |
| TEDS | 4578 | 9.02 | 0.28 | 8.50 | 10.46 | 2123 | 9.02 | 0.28 | 8.50 | 10.46 | 2455 | 9.01 | 0.28 | 8.50 | 10.46 |

Note: N = sample size, SD = standard deviation, Min = minimum age, Max = Maximum age. *No individual age data available for CATSS cohort

**Supplementary Table 3. Descriptive statistics for childhood measures**

|  | | | All | | | | | Males | | | | | Females | | | | |
| --- | --- | --- | --- | --- | --- | --- | --- | --- | --- | --- | --- | --- | --- | --- | --- | --- | --- |
| Cohort | **Scale** | **Measure** | **N** | **Mean** | **SD** | **Min** | **Max** | **N** | **Mean** | **SD** | **Min** | **Max** | **N** | **Mean** | **SD** | **Min** | **Max** |
| ALSPAC | SDQ | ADHD symptoms | 4827 | 2.83 | 2.20 | 0.00 | 10.00 | 2455 | 3.21 | 2.32 | 0.00 | 10.00 | 2372 | 2.43 | 1.99 | 0.00 | 10.00 |
|  |  | Internalizing problems | 4896 | 1.42 | 1.68 | 0.00 | 10.00 | 2473 | 1.29 | 1.63 | 0.00 | 10.00 | 2423 | 1.56 | 1.72 | 0.00 | 10.00 |
|  |  | Social problems | 4688 | 1.03 | 1.42 | 0.00 | 10.00 | 2349 | 1.11 | 1.52 | 0.00 | 10.00 | 2339 | 0.94 | 1.31 | 0.00 | 10.00 |
| CATSS | A-TAC | ADHD symptoms | 7281 | 1.78 | 2.81 | 0.00 | 19.00 | 3633 | 2.17 | 3.12 | 0.00 | 19.00 | 3648 | 1.40 | 2.40 | 0.00 | 19.00 |
|  | SCARED | Internalizing problems | 4277 | 4.83 | 5.97 | 0.00 | 66.00 | 2112 | 4.42 | 5.65 | 0.00 | 66.00 | 2165 | 5.22 | 6.24 | 0.00 | 50.00 |
| NTR | CBCL | ADHD symptoms | 3647 | 3.03 | 3.24 | 0.00 | 18.00 | 1723 | 3.51 | 3.40 | 0.00 | 15.00 | 1924 | 2.61 | 3.03 | 0.00 | 18.00 |
|  |  | Internalizing problems | 3604 | 4.90 | 5.42 | 0.00 | 45.00 | 1701 | 4.63 | 5.28 | 0.00 | 41.00 | 1903 | 5.15 | 5.53 | 0.00 | 45.00 |
|  |  | Social problems | 3642 | 2.09 | 2.59 | 0.00 | 18.00 | 1720 | 2.18 | 2.62 | 0.00 | 17.00 | 1922 | 2.01 | 2.57 | 0.00 | 18.00 |
| TEDS | SDQ | ADHD symptoms | 4577 | 3.23 | 2.40 | 0.00 | 10.00 | 2123 | 3.75 | 2.57 | 0.00 | 10.00 | 2454 | 2.77 | 2.13 | 0.00 | 10.00 |
|  |  | Internalizing problems | 4578 | 1.72 | 1.89 | 0.00 | 10.00 | 2123 | 1.54 | 1.83 | 0.00 | 10.00 | 2455 | 1.88 | 1.93 | 0.00 | 10.00 |
|  |  | Social problems | 4578 | 1.03 | 1.52 | 0.00 | 10.00 | 2123 | 1.13 | 1.62 | 0.00 | 10.00 | 2455 | 0.94 | 1.41 | 0.00 | 9.00 |

Note: N = sample size, SD = standard deviation, Min = minimum score, Max = Maximum score, A-TAC, Autism-Tics, AD/HD and other comorbidities inventory; CBCL, Child Behaviour Checklist; SDQ, Strength and Difficulties Questionnaire; SMFQ, Short Mood and Feelings Questionnaire; SCARED, Screen for Child Anxiety Related Emotional Disorders.

References

1. Boyd A, Golding J, Macleod J, et al. Cohort Profile: the 'children of the 90s'--the index offspring of the Avon Longitudinal Study of Parents and Children. *International journal of epidemiology*. 2013;42(1):111-127. doi:10.1093/ije/dys064

2. Fraser A, Macdonald-Wallis C, Tilling K, et al. Cohort Profile: the Avon Longitudinal Study of Parents and Children: ALSPAC mothers cohort. *Int J Epidemiol*. Feb 2013;42(1):97-110. doi:10.1093/ije/dys066

3. Northstone K, Lewcock M, Groom A, et al. The Avon Longitudinal Study of Parents and Children (ALSPAC): an update on the enrolled sample of index children in 2019. *Wellcome Open Research*. 2019;4doi:10.12688/wellcomeopenres.15132.1

4. Anckarsäter H, Lundström S, Kollberg L, et al. The Child and Adolescent Twin Study in Sweden (CATSS). *Twin Research and Human Genetics*. 2012;14(6):495-508. doi:10.1375/twin.14.6.495

5. Van Beijsterveldt CE, Groen-Blokhuis M, Hottenga JJ, et al. The Young Netherlands Twin Register (YNTR): longitudinal twin and family studies in over 70,000 children. *Twin Research and Human Genetics*. 2013;16(1):252-267.

6. Rimfeld K, Malanchini M, Spargo T, et al. Twins Early Development Study: A Genetically Sensitive Investigation into Behavioral and Cognitive Development from Infancy to Emerging Adulthood. *Twin Research and Human Genetics*. 2019;22(6):508-513. doi:10.1017/thg.2019.56

7. Wray NR, Ripke S, Mattheisen M, et al. Genome-wide association analyses identify 44 risk variants and refine the genetic architecture of major depression. *Nat Genet*. May 2018;50(5):668-681. doi:10.1038/s41588-018-0090-3

8. Stahl EA, Breen G, Forstner AJ, et al. Genome-wide association study identifies 30 loci associated with bipolar disorder. *Nature Genetics*. 2019/05/01 2019;51(5):793-803. doi:10.1038/s41588-019-0397-8

9. Okbay A, Baselmans BML, De Neve J-E, et al. Genetic variants associated with subjective well-being, depressive symptoms, and neuroticism identified through genome-wide analyses. Article. *Nat Genet*. 06//print 2016;48(6):624-633. doi:10.1038/ng.3552

<http://www.nature.com/ng/journal/v48/n6/abs/ng.3552.html#supplementary-information>

10. Hammerschlag AR, Stringer S, de Leeuw CA, et al. Genome-wide association analysis of insomnia complaints identifies risk genes and genetic overlap with psychiatric and metabolic traits. Article. *Nature Genetics*. 06/12/online 2017;49:1584. doi:10.1038/ng.3888

<https://www.nature.com/articles/ng.3888#supplementary-information>

11. Lee JJ, Wedow R, Okbay A, et al. Gene discovery and polygenic prediction from a genome-wide association study of educational attainment in 1.1 million individuals. *Nature Genetics*. 2018/08/01 2018;50(8):1112-1121. doi:10.1038/s41588-018-0147-3

12. Yengo L, Sidorenko J, Kemper KE, et al. Meta-analysis of genome-wide association studies for height and body mass index in approximately 700000 individuals of European ancestry. *Hum Mol Genet*. Oct 15 2018;27(20):3641-3649. doi:10.1093/hmg/ddy271
